# Sarmentosamide, an Anti-Aging Compound from a Marine-Derived *Streptomyces* sp. APmarine042

**DOI:** 10.3390/md18090463

**Published:** 2020-09-10

**Authors:** Eun-Soo Lee, Eun-Young Lee, Jisoo Yoon, Ahreum Hong, Sang-Jip Nam, Jaeyoung Ko

**Affiliations:** 1 Amorepacific Corporation R&D Center, Yongin 17074, Korea; soopian82@gmail.com; 2 Department of Chemistry and Nanoscience, Ewha Womans University, Seoul 03760, Korea; younglee0124@naver.com (E.-Y.L.); jisoo87@naver.com (J.Y.); 3 Graduate School of Industrial Pharmaceutical Sciences, Ewha Womans University, Seoul 03760, Korea; lyzenne@naver.com

**Keywords:** sarmentosamide, *Streptomyces* sp. APmarine042, anti-aging, MMP-1, UVB, TNF-α

## Abstract

Many bioactive materials have been isolated from marine microorganisms, including alkaloids, peptides, lipids, mycosporine-like amino acids, glycosides, and isoprenoids. Some of these compounds have great potential in the cosmetic industry due to their photo-protective, anti-aging, and anti-oxidant activities. In this study, sarmentosamide (**1**) was isolated from marine-derived *Streptomyces* sp. APmarine042, after which its capacity to decrease skin aging was examined in-vitro. Sarmentosamide (**1**) was found to significantly reduce UVB-induced matrix metalloproteinase-1 (MMP-1) expression in normal human dermal fibroblasts (NHDFs) by inhibiting the extracellular signal-regulated kinase (ERK) and the c-Jun N-terminal kinase (JNK) phosphorylation, which are regulatory pathways upstream of MMP-1 transcription. Additionally, we confirmed that sarmentosamide (**1**) decreased tumor necrosis factor-alpha (TNF-α), induced MMP-1 secretion in NHDFs, and exhibited free-radical scavenging activity, as demonstrated by 2,2-diphenyl-1-picrylhydrazyl (DPPH) assay. Therefore, our study suggests that sarmentosamide (**1**) could be a promising anti-aging agent that acts via the downregulation of MMP-1 expression.

## 1. Introduction

Skin aging is induced by two main processes, which result from intrinsic and extrinsic factors. Extrinsic aging is primarily caused by exposure to environmental factors such as air pollution [1] and ultraviolet (UV) radiation [2]. UV light is composed of UVA (315–400 nm), UVB (280–315 nm), and UVC (200–280 nm), of which UVB rays are known to penetrate the epidermis and contribute to skin photoaging [3]. UVB-induced skin photoaging causes DNA damage [4] and reactive oxygen species (ROS) generation [5] and disrupts the extracellular matrix [6]. Intrinsic aging is caused by the natural consequences of physiological change, such as genetic factors, hormones, and metabolic processes [7]. Moreover, inflammatory cytokines, such as tumor necrosis factor-alpha (TNFα), interleukin 1 alpha/beta (IL-1α/β), and interleukin 6 (IL-6), are chronically increased with age. In particular, tumor necrosis factor-α(TNFα) is known to accelerate the degradation of extracellular matrix (ECM) components via upregulating expression and activity of matrix metalloproteinases (MMPs) in aged skin [8]. MMP-1 is mainly released by fibroblasts in the dermis, and secreted MMP-1 is responsible for the degradation of dermal collagen, which supports skin structure and function. Therefore, modulation of MMP-1 expression in dermal fibroblasts could be a promising target for the development of anti-aging cosmetic ingredients.

Previous studies have reported that UVB radiation induces ROS (reactive oxygen species) production and leads to the activation of intracellular signaling pathways and transcription factors (e.g., AP-1, NF-κB) [9]. These transcription factors are regulated by mitogen-activated protein kinases (MAPKs), which induce MMP-1 and proinflammatory cytokines. There are three distinct MAPK families involved in UVB/ROS stimuli: extracellular signal-regulated kinases (ERKs), c-Jun N-terminal kinase (JNK), and p38.

Our study identified a *Streptomyces* sp. APmarine042 extract that exhibited a potent inhibitory effect on MMP-1 expression. Afterward, an MMP-1 inhibitory compound was identified in this *Streptomyces* sp. APmarine042 extract, and its chemical structure was identified via NMR analyses as sarmentosamide (**1**), a compound with a hexadienamide moiety [10]. We then further investigated whether this compound inhibited MMP-1 expression in UVB-irradiated and TNFα-induced human dermal fibroblasts as well as its role in the regulation of underlying signaling pathways. Additionally, we examined the antioxidant activity of sarmentosamide (**1**) via a 2,2-diphenyl-1-picrylhydrazyl (DPPH) radical scavenging assay.

## 2. Results and Discussion

### 2.1. Sarmentosamide (1) Isolation and Identification

**Figure 1:**
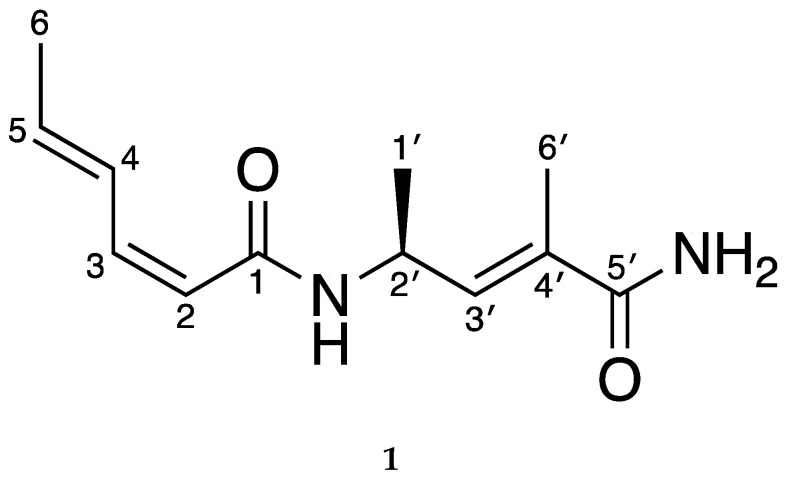
Chemical structure of sarmentosamide (**1**).

Sarmentosamide (**1**) was isolated through a combination of HPLC-UV and bioactivity-guided isolation. Based on the comparison between the NMR data (Supplementary Materials) of the compound identified in this study (hereinafter referred to as “compound **1**”) to those of previously reported compounds, compound **1** was identified as sarmentosamide. The optical rotation value of compound **1** was also consistent with previous reports, which further supported that compound **1** was sarmentosamide (Figure 1).

### 2.2. Cytotoxic Effects of Sarmentosamide (1) on Human Dermal Fibroblasts

**Figure 2:**
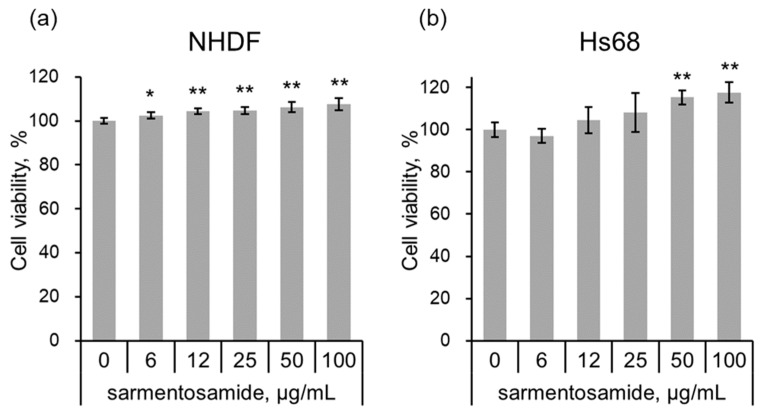
Cell viability test of sarmentosamide on fibroblasts. (**a**,**b**) The cell viability of sarmentosamide-treated normal human dermal fibroblasts (NHDF) (**a**) and human foreskin fibroblast cells (Hs68) (**b**) cells at 24 h was measured by CCK (cell counting kit) assay. * *p* < 0.05; ** *p* < 0.01 (vs. untreated group).

To investigate the cytotoxic effects of sarmentosamide, normal human dermal fibroblasts (NHDF) or human foreskin fibroblast cells (Hs68) were treated with sarmentosamide at different concentrations (6, 12, 25, 50, 100 µg/mL) for 24 h, after which the cell counting kit-8 (CCK-8) assay was performed to quantify cell viability. Water-soluble tetrazolium salt (WST-8) in CCK-8 solution was reduced by dehydrogenase activities in cells to give a yellow-color formazan dye, the amount of which was proportional to the number of living cells. As shown in Figure 2, sarmentosamide did not cause any observable cytotoxic effects in either NHDF or Hs68 cells at concentrations ranging from 0–100 µg/mL.

### 2.3. Sarmentosamide (1) Inhibits MMP-1 Expression and Secretion in UVB-Irradiated NHDF Cells

**Figure 3:**
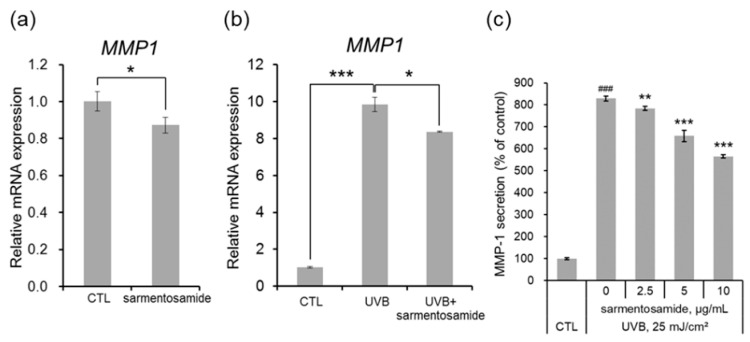
Effect of sarmentosamide on metalloproteinase-1 (MMP-1) expression in UVB-irradiated NHDFs. (**a**,**b**) The relative mRNA levels of MMP-1 in NHDF cells irradiated UVB (25 mJ/cm^2^) or treated with sarmentosamide (2.5 µg/mL) for 16 h. RPLP0 gene was used as an internal control. * *p* < 0.05; *** *p* < 0.001 (**c**) After UVB irradiation (25 mJ/cm^2^), sarmentosamide in serum-free media was treated for 48 h, and then the relative secretion levels of MMP-1 into cell culture media were analyzed by ELISA. ### *p* < 0.001 (vs. non-UVB control group); ** *p* < 0.01; *** *p* < 0.001 (UVB-irradiated control group).

Next, NHDFs in the absence and the presence of UVB radiation (25 mJ/cm^2^) were treated with sarmentosamide (2.5 µg/mL) for short periods, and relative MMP-1 mRNA expression levels were quantified via quantitative real-time PCR analysis. Sarmentosamide was found to downregulate MMP-1 mRNA levels by up to 16% and 17% in the absence (Figure 3a) and the presence (Figure 3b) of UVB, respectively. UVB radiation, a major extrinsic skin aging risk factor, can penetrate the epidermis and reach the upper dermis, resulting in the generation of reactive oxygen species (ROS), MMP production, and collagen synthesis downregulation. Therefore, our study sought to investigate whether sarmentosamide inhibited MMP-1 secretion in UVB-irradiated NHDF cells. The secretion levels of MMP-1 in cell culture media were measured via ELISA, and the relative amounts of MMP-1 secretion were normalized to total protein in whole cell lysates, which were measured via a bicinchoninic acid (BCA) protein quantification assay. UVB irradiation (25 mJ/cm^2^) remarkably increased MMP-1 secretion (8.3-fold) compared to the non-UVB-irradiated group (Figure 3c) and sarmentosamide inhibited MMP-1 secretion in UVB-irradiated NHDF cells in a dose-dependent manner.

### 2.4. Sarmentosamide (1) Downregulates JNK and ERK Phosphorylation in UVB-Irradiated NHDF Cells

**Figure 4:**
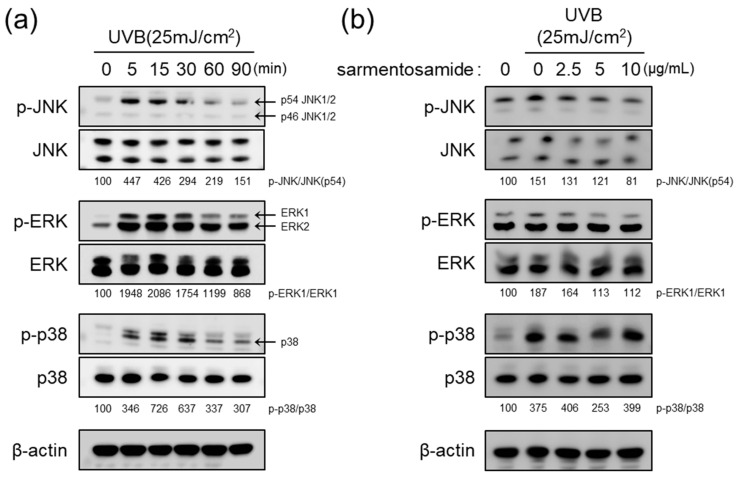
Inhibitory effect of sarmentosamide on mitogen-activated protein kinases (MAPK) signaling pathway activation in UVB-irradiated NHDF cells. (**a**) Western blot analysis of MAPK (c-Jun N-terminal kinase (JNK), extracellular signal-regulated kinase (ERK), and p38) signaling pathway time-kinetics after UVB (25 mJ/cm^2^) exposure. (**b**) NHDFs were pretreated with sarmentosamide at the different concentrations for 24 h. The cells were then harvested for western blot analysis at 15 min post-UVB irradiation (25 mJ/cm^2^). Densitometric quantification results for relative ratios of band intensities of phosphor-MAPK/total-MAPK were inserted in figure.

UVB radiation induces MMP-1 gene transcription by triggering the MAPK signaling pathway via the phosphorylation of JNK, ERK, and p38 signaling cascades [3]. In the NHDF cells used in this study, we confirmed that UVB induced JNK, ERK, and p38 phosphorylation in a time dependent manner. As shown in Figure 4a, MAPK phosphorylation levels reached a peak at 15 min. Therefore, to examine whether sarmentosamide attenuates UVB induced MAPK phosphorylation, NHDFs were pretreated with sarmentosamide for 24 h and irradiated with UVB (25 mJ/cm^2^). After 15 min post-irradiation, the phosphorylation levels of three different MAPK proteins were measured via western blot. Our results demonstrated that sarmentosamide decreased the levels of phospho-JNK and phospho-ERK but not phospho-p38 in a dose-dependent manner (Figure 4b).

### 2.5. Sarmentosamide (1) Inhibits MMP-1 Secretion in TNFα-Treated NHDF Cells

**Figure 5:**
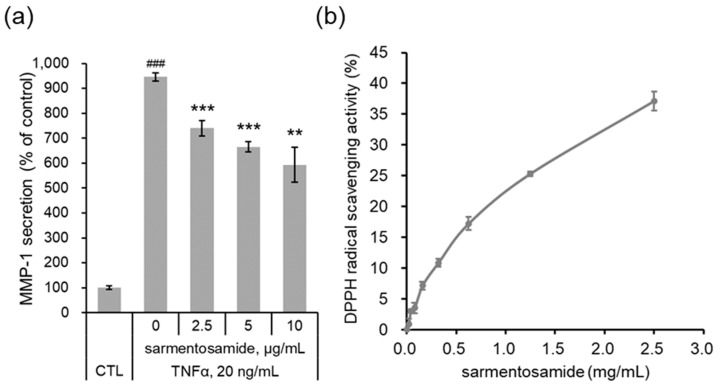
Protective effect of sarmentosamide on tumor necrosis factor-alpha (TNFα)-induced skin aging. (**a**) Cells were co-treated with TNFα (20 ng/mL) and sarmentosamide in serum-free media for 48 h, after which the relative secretion levels of MMP-1 in cell culture media were analyzed via ELISA. ### *p* < 0.001 (relative to the TNFα-untreated control group); ** *p* < 0.01; *** *p* < 0.001 (TNFα-treated control group). (**b**) The free radical scavenging activity of sarmentosamide was measured via the 2,2-diphenyl-1-picrylhydrazyl (DPPH) assay.

In addition to external stimuli (e.g., UV irradiation), intrinsic stimuli such as inflammatory cytokines can induce MMP-1 production. The proinflammatory cytokine TNFα is known to induce MMP-1 expression in dermal fibroblasts [8]. Therefore, we further investigated whether sarmentosamide downregulated MMP-1 secretion in TNFα-induced NHDF cells. As shown in Figure 5a, TNFα (20 ng/mL) upregulated MMP-1 secretion in NHDFs by up to 9.4-fold compared to the untreated control group. Treatment with sarmentosamide for 48 h downregulated TNFα-induced MMP-1 secretion in a dose-dependent manner. Specifically, the inhibitions of TNFα-induced MMP-1 secretion by sarmentosamide treatment at 2.5, 5, and 10 µg/mL were 24%, 33%, and 42%, respectively.

### 2.6. Antioxidant Effect of Sarmentosamide (1) on DPPH Radical Scavenging

Reactive oxygen species (ROS) activate AP-1 [11] and NF-κB [12] under inflammatory conditions and disturb the cellular redox balance. Upon exposure to the inflammatory cytokine TNFα, cells not only activate MAPK signaling but also intracellular ROS production, resulting in a positive feedback that accelerates AP-1- and NF-κB-mediated MMP-1 expression [13]. Therefore, reducing ROS generation mitigates TNFα-mediated skin aging. As shown in Figure 5b, upon evaluating the antioxidant capacity of sarmentosamide via a DPPH assay, this compound was found to possess a remarkable radical scavenging activity.

## 3. Materials and Methods

### 3.1. General Experimental Procedures

Extracts isolation was conducted by binary HPLC (high-performance liquid chromatography) pump (HPLC WATERS™ 600, Milford, MA, USA) coupled with a WATERS 996 photodiode array (PDA) UV/Vis detector and the reversed-phase semi-prep HPLC isolation condition (Phenomenex Luna 5µ C18 column, 250 mm × 10 mm, 5 µm, eluting with 40% CH_3_CN at flow rate 2.0 mL/min). ESI (electrospray ionization) low-resolution LC-MS data were obtained with an Agilent Technologies 6120 quadrupole mass system (Agilent Technologies, Santa Clara, CA, USA) coupled with an Agilent Technologies 1260 series HPLC with a reversed-phase Phenomenex luna C18 column (4.6 mm × 100 mm, 5 µm) at a low flow rate of 1.0 mL/min. NMR spectra were acquired by Bruker Avance 300 MHz and 150 MHz spectrometers (Bruker Biospin Group, Karlsruhe, Germany) using methanol-*d*_4_ as a solvent, which was purchased from Cambridge Isotope Laboratories, Inc. (Tewksbury, MA, USA). For extract fractionation, first grade solvents were acquired from Dae-Jung chemicals & Metals Co. Ltd. For LC-MS and HPLC analyses, HPLC-grade solvents were provided from J.T.Baker. and Dae-Jung chemicals & Metals Co. Ltd. (Sheung-Si, Korea). 

### 3.2. Bacterial Strain

The APmarine042 strain was isolated from marine-derived sediment obtained in choupori sinan goon, Jeollannam-do, South Korea, in 2013. Based on 16 s rRNA gene sequence analyses, the strain was identified as a member of the genus *Streptomyces*, sharing the highest identity (99.9%) with *Streptomyces coelicolor*.

### 3.3. Fermentation and Extraction

*Streptomyces* sp. APmarine042 was cultured in 40 L and 2.5 L Ultra Yield Flasks, each containing 1 L of the seawater-based medium (10 g/L of soluble starch, 4 g/L of yeas, 2 g/L of peptone, 10 g/L of CaCO_3_, 20 g/L of KBr, 8 g/L of Fe_2_(SO_4_)_3_·4H_2_O dissolved in 750 mL natural seawater and 250 mL of distilled water at 25 °C with shaking at 150 rpm). After seven days cultivation, the aqueous layer (1 L) was extracted with an equal volume of ethyl acetate (EtOAc). The EtOAc-soluble layers were then combined and concentrated under reduced pressure to yield 1.5 g *Streptomyces* sp. APmarine042 extracts.

### 3.4. Isolation

The 1.5 g *Streptomyces.* sp. APmarine042 were fractionated via flash column chromatography on silica gel eluted with CH_2_Cl_2_/CH_3_OH (100/0, 100/1, 100/5, 100/10, 100/20, 100/100, and 0/100 *v/v*, each of 200 mL) to obtain eight sub-fractions (Fraction 1–Fraction 8). The fifth fraction (Fraction 4) was further fractionated by reversed-phase HPLC (Phenomenex Luna C-18 (2), 250 × 100 mm, 2.0 mL/min, 5 μm, 100 Å, UV = 254 nm) using an isocratic condition 50% CH_3_CN in H_2_O to obtain sarmentosamide (17.0 mg).

Sarmentosamide (**1**): [α]_D_ = −168° (C 0.05, CH_3_OH); ^1^H NMR (CD_3_OD, 300 MHz): δ_H_ 7.43 (1H, dd, *J* = 15.1, 11.4 Hz, H-4), 6.42 (1H, dd, *J* = 11.8, 11.4 Hz, H-3), 6.27, (1H, dq, *J* = 8.8, 1.5 Hz, H-3′), 6.01 (1H, qd, *J* = 15.1, 6.8 Hz, H-5), 5.59 (dd, *J* = 11.8, 0.7 Hz, H-2), 4.80 (m, H-2′), δ_H_ 1.95 (m, H-6′), 1.85 (3H, dd, *J* = 6.8, 1.7 Hz, H-6), 1.26 (3H, d, *J* = 7.0 Hz, H-1′); ^13^C NMR (150 MHz, CD_3_OD): δ_C_ 172.6 (qC, C-5′), 166.8 (qC, C-1), 141.2 (CH, C-3), 137.7 (CH, C-3′), 137.6 (CH, C-5), 130.9 (qC, C-4′), 128.9 (CH, C-4), 117.8 (CH, C-2), 42.9 (CH, C-2′), 19.2 (CH_3_, C-1′), 17.3 (CH_3_, C-6), 11.9 (CH_3_, C-6′); LR-MS *m/z*: 223.14 [M + H]^+^.

### 3.5. Cell Culture and Viability Assay

Normal human dermal fibroblasts, neonatal (NHDFs), and human foreskin fibroblast cell line (Hs68) were purchased from Thermo Fisher Scientific (#C-004 − 5C, Waltham, MA, USA) and American Type Culture Collection (#CRL-1635, Manassas, VA, USA), respectively, and cultured in Dulbecco’s modified Eagle’s medium (DMEM, #12−604F, Lonza, Walkersville, MD, USA) containing 10% fetal bovine serum (#16000–044, Thermo Fisher Scientific), 100 U/mL potassium penicillin, and 100 mg/mL streptomycin sulfate (#17 − 602E, Lonza) at 37 °C in a humidified 5% CO_2_ incubator. NHDFs were treated with serial sarmentosamide concentrations for 24 h, after which the CCK-8 reagent (Dojindo Bio., Japan) was used to quantify cell viability according to the manufacturer’s instructions (*n* = 4 per group).

### 3.6. MMP-1 ELISA (Enzyme-Linked Immunosorbent Assay)

NHDFs were pretreated with serum-free DMEM media for 18 h, then exposed to 25 mJ/cm^2^ UVB (average intensity: 2.7 mJ/cm^2^) or treated with 20 ng/mL of TNFα and then co-treated with sarmentosamide at the different concentrations (2.5, 5, 10 µg/mL) in a serum-free DMEM media for an additional 48 h. The amounts of secreted MMP-1 in the culture medium were quantified by an MMP-1 ELISA kit (R&D system, #DY901) according to the manufacturer’s protocol. The final MMP-1 secretion results were normalized to the protein amounts in whole cell lysates, which were measured by a BCA protein quantification assay (*n* = 3 per group).

### 3.7. Western Blots

Cell lysates aliquots were subjected to western blot assay as previously described [3]. Membranes were incubated with specific antibodies against JNK (#9252, Cell Signaling Technology, Beverly, MA, USA), *p*-JNK (#9251s), ERK (#4695 s), *p*-ERK (4370 s), p38 (#9212 s), *p*-p38 (#4511 s), and β-actin (#sc-1616, Santa Cruz Biotechnology, Dallas, TX, USA) in Tris-buffered saline (TBS) buffer containing 5% bovine serum albumin (BSA) and 0.1% Tween-20 at 4 °C overnight. After washing, the membranes were allowed to react with peroxidase-conjugated secondary antibodies in TBS buffer containing 5% BSA and 0.1% Tween-20 at room temperature for 1 h. Chemiluminescent signals were detected with an enhanced chemiluminescence (ECL) substrate (#RPN2232, GE Healthcare, UK) and visualized with a chemiluminescence detection device (Fuji Film, Tokyo, Japan).

### 3.8. DPPH Radical Scavenging Assay

The 2,2-diphenyl-1-picrylhydrazyl (DPPH) was purchased from Sigma Aldrich (#D9132). The DPPH radical scavenging assay was performed according to the previous reports with minor modification [14]. A total of 100 µM of DPPH solution was freshly prepared with 100% ethanol. Then, 10 µL of sarmentosamide solutions at the different concentrations were loaded onto 96-well plates, followed by 190 µL of the DPPH solution. The 96-well plates were then incubated in a dark chamber at 37 °C for 30 min. Absorbances were then measured at 517 nm with a microplate reader (Biotek, Synergy HTX Multi-Mode Reader). DPPH radical scavenging activity was calculated with the following formula:

$$\mathrm{DPPH}\;\mathrm{radical}\;\mathrm{scavenging}\;\mathrm{activity}\left(\%\right)=\frac{\mathrm{Abs}517\mathrm{nm}\;\mathrm{of}\;\mathrm{control}-\mathrm{Abs}517\mathrm{nm}\;\mathrm{of}\;\mathrm{sample}}{\mathrm{Abs}517\mathrm{nm}\;\mathrm{of}\;\mathrm{control}}\;\times 100$$

where Abs517nm of control is the absorbance of the 190 µL of DPPH mixed with 10 µL of ethanol solution, and Abs517nm of sample is absorbance of the 190 µL of DPPH mixed with 10 µL of sarmentosamide solution.

## 4. Conclusions

In conclusion, an extract library of 250 marine microorganisms was screened to identify novel anti-aging compounds, of which one active *Streptomyces* sp. APmarine042 extract was selected. We then characterized the chemical structure of an active compound in the *Streptomyces* sp. APmarine042 extract using 1D and 2D NMR spectra and identified the compounds as sarmentosamide. Furthermore, we demonstrated that sarmentosamide significantly reduced UVB-induced MMP-1 expression and secretion in NHDFs by inhibiting ERK and JNK phosphorylation. Moreover, we confirmed that sarmentosamide decreased TNFα-induced MMP-1 secretion in NHDFs and assessed its free-radical scavenging activity via the DPPH assay. Our results indicated that sarmentosamide could be a promising skin anti-aging agent that acts via the downregulation of MMP-1 expression.

## Supplementary Materials

The following are available online at https://www.mdpi.com/1660-3397/18/9/463/s1, Figure S1: ^1^H NMR spectrum (300 MHz, CD_3_OD) of sarmentosamide, Figure S2: ^13^C NMR spectrum (150 MHz, CD_3_OD) of sarmentosamide.
