# Supplementary material for: Sarmentosamide, an Anti-Aging Compound from a Marine-Derived Streptomyces sp. APmarine042
Source: Mar Drugs. 2020 Sep 10;18(9):463. doi: 10.3390/md18090463 (PMC7551700; doi:10.3390/md18090463)
Supplement: Supplementary file 1 [file marinedrugs-18-00463-s001.pdf]

2020-08-08 23:20/AP490610

Supporting Information for

Sarmentosamide, an Anti-aging Compound from a Marine-Derived *Streptomyces* sp. APmarine042

Eun-Soo Lee [1,†] , Eun-Young Lee [2,†], Jisoo Yoon [2], Ahreum Hong [3], Sang-Jip Nam[2,*], and Jaeyoung Ko [1,*]

[1] Amorepacific Corporation R&D Center, Yongin, Republic of Korea; soopian82@gmail.com (E-S.L.);jaeyoungko@amorepacific.com (J.K.)
[2] Department of Chemistry and Nanoscience, Ewha Womans University, Seoul 03760, Korea; younglee0124@naver.com (E.-Y.L.);jisoo87@naver.com (J.Y.); sjnam@ewha.ac.kr (S.-J.N)
[3] Graduate School of Industrial Pharmaceutical Sciences, Ewha Womans University, Seoul 03760, Republic of Korea; lyzenne@naver.com (A.H.)
[†] Both authors contributed equally to this work.
* Correspondence: jaeyoungko@amorepacific.com; Tel.: +82-31-280-5928 (J.K); sjnam@ewha.ac.kr; Tel.: +82-2-3277-6805 (S.-J.N)

AMOREPACIFIC 고재영
AMOREPACIFIC 고재영
본 문서는 저작권, 영업비밀을 포함한 모든 권리가 AMOREPACIFIC에 있으며, 무단으로 유출할 때에는 회사 규정에 의해 징계 및 법적 처벌을 받을 수 있습니다.
Copyrightⓒ 2020 AMOREPACIFIC All Rights Reserved.

2020-08-08 23:20/AP490610

Table of Contents

Figure S1 ^{1}H NMR spectrum (300 MHz, CD_3OD) of sarmentosamdie (**1**) ········· **S3**

Figure S2 ^{13}C NMR spectrum (150 MHz, CD_3OD) of sarmentosamdie (**1**) ········· **S4**

AMOREPACIFIC 고재영

AMOREPACIFIC 고재영

본 문서는 저작권, 영업비밀을 포함한 모든 권리가 AMOREPACIFIC에 있으며, 무단으로 유출할 때에는 회사 규정에 의해 징계 및 법적 처벌을 받을 수 있습니다.
Copyrightⓒ 2020 AMOREPACIFIC All Rights Reserved.

2020-08-08 23:20/AP490610

Figure S1 ^{1}H NMR spectrum (300 MHz, CD_3OD) of sarmentosamdie (**1**)

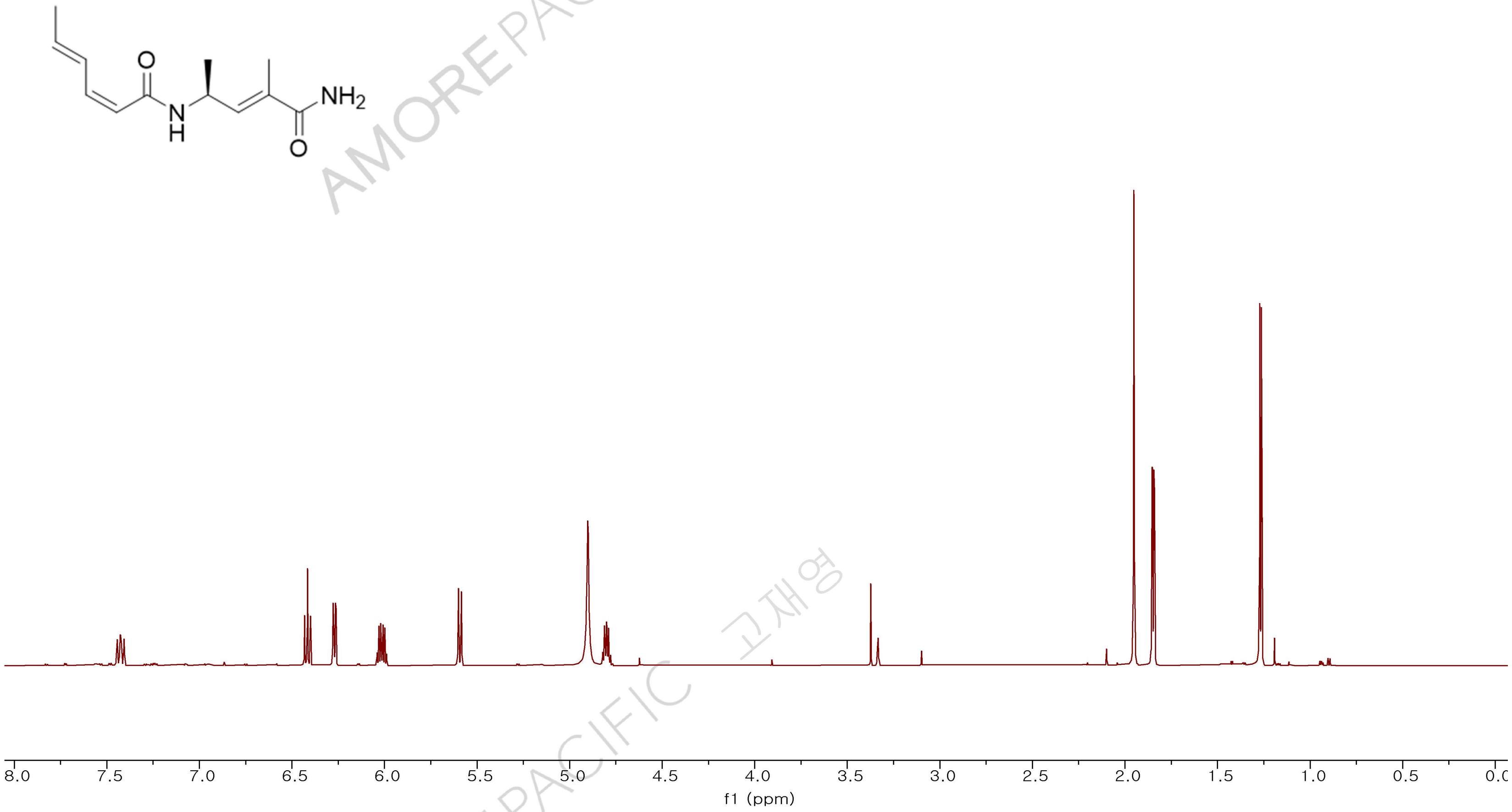

AMOREPACIFIC 고재영
AMOREPACIFIC 고재영

본 문서는 저작권, 영업비밀을 포함한 모든 권리가 AMOREPACIFIC에 있으며, 무단으로 유출할 때에는 회사 규정에 의해 징계 및 법적 처벌을 받을 수 있습니다.
Copyrightⓒ 2020 AMOREPACIFIC All Rights Reserved.

2020-08-08 23:20/AP490610

Figure S2 ^{13}C NMR spectrum (150 MHz, CD_3OD) of sarmentosamide (**1**)

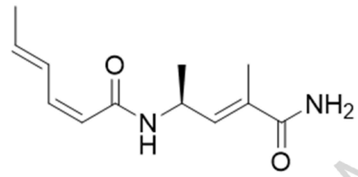

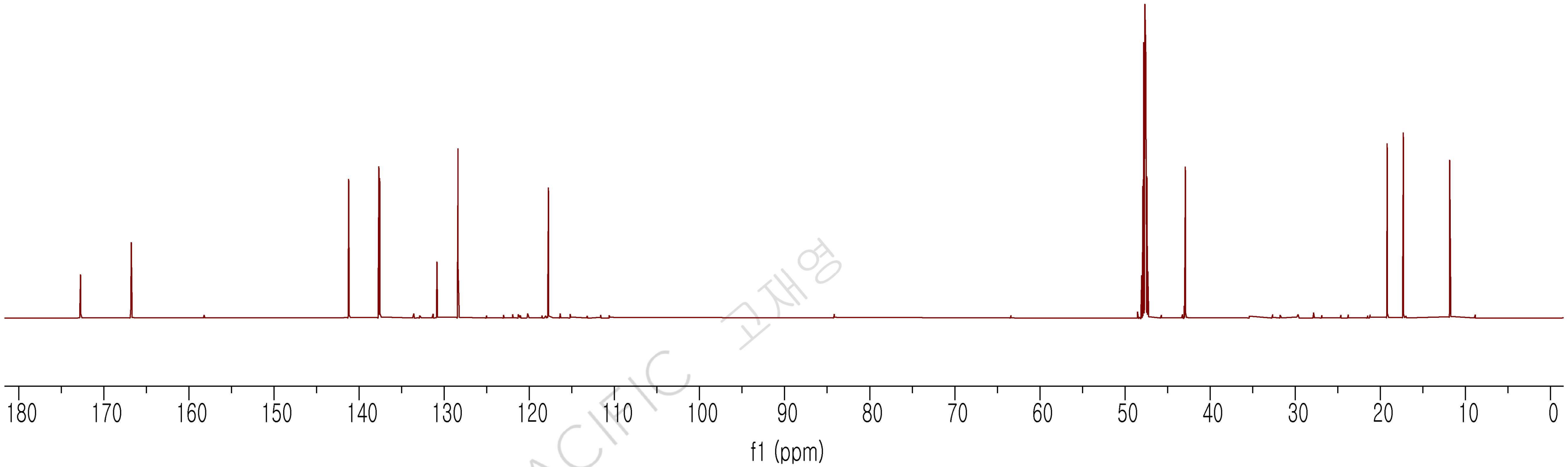

AMOREPACIFIC 고재영
AMOREPACIFIC 고재영
본 문서는 저작권, 영업비밀을 포함한 모든 권리가 AMOREPACIFIC에 있으며, 무단으로 유출할 때에는 회사 규정에 의해 징계 및 법적 처벌을 받을 수 있습니다.
Copyrightⓒ 2020 AMOREPACIFIC All Rights Reserved.
